# Emotion Words’ Effect on Visual Awareness and Attention of Emotional Faces

**DOI:** 10.3389/fpsyg.2019.02896

**Published:** 2020-01-15

**Authors:** Jennifer M. B. Fugate, Cameron MacDonald, Aminda J. O’Hare

**Affiliations:** ^1^Department of Psychology, University of Massachusetts Dartmouth, Dartmouth, MA, United States; ^2^Department of Psychology, Weber State University, Ogden, UT, United States

**Keywords:** emotion, visual awareness, visual attention, emotional faces, semantic priming, emotion words, binocular rivalry, divided visual field

## Abstract

To explore whether the meaning of a word changes visual processing of emotional faces (i.e., visual awareness and visual attention), we performed two complementary studies. In Experiment 1, we presented participants with emotion and control words and then tracked their visual awareness for two competing emotional faces using a binocular rivalry paradigm. Participants experienced the emotional face congruent with the emotion word for longer than a word-incongruent emotional face, as would be expected if the word was biasing awareness toward the (unseen) face. In Experiment 2, we similarly presented participants with emotion and control words prior to presenting emotional faces using a divided visual field paradigm. Emotion words were congruent with either the emotional face in the right or left visual field. After the presentation of faces, participants saw a dot in either the left or right visual field. Participants were slower to identify the location of the dot when it appeared in the same visual field as the emotional face congruent with the emotion word. The effect was limited to the left hemisphere (RVF), as would be expected for linguistic integration of the word with the face. Since the task was not linguistic, but rather a simple dot-probe task, participants were slower in their responses under these conditions because they likely had to disengage from the additional linguistic processing caused by the word-face integration. These findings indicate that emotion words bias visual awareness for congruent emotional faces, as well as shift attention toward congruent emotional faces.

## Introduction

In everyday life, people use words to communicate what they see in the world around them, usually without considering that those same words might actually be shaping what they see. Accumulating evidence indicates that words are important, if not necessary, for emotion perception (for reviews, see Barrett et al., 2007; Fugate and Barrett, 2014; Lindquist et al., 2015, 2016). Whether emotion words affect visual awareness and attention for emotional information has not been thoroughly investigated. Before turning to this question, we review studies in which emotion words have been shown to affect varying, later stages of processing.

In one landmark study, participants had a difficult time deciding whether two emotional faces matched when an emotion word was satiated (repeating the emotion word until the word loses meaning) compared to primed (Lindquist et al., 2006). For example, when two *scowling* faces were shown side-by-side and participants were satiated on the word “anger” (matching the content of the faces), participants were slower and less accurate to determine the pair matched in emotional content compared to when the word “anger” was primed. In another experiment using a similar semantic satiation manipulation, participants saw an emotional face and were asked to encode it. Later in the experiment, participants saw the same faces, but without undergoing satiation. Participants did not show repetition priming for the emotional faces when the face was re-presented with access to the emotion word (Gendron et al., 2012). The fact that participants did not show this phenomenon suggested that when the same face was seen in the presence (vs. absence) of emotion words, the visual system did not “recognize” the face. In a more recent study, participants were asked which one of several emotional faces was the target face. Participants showed greater bias (incorrect answers) when they were forced to first label the target face (e.g., *relaxing*) with a partially incongruent word (e.g., *smiling*) compared to when they were able to label the word with a fully congruent emotion word (e.g., “calm”). That is, participants’ perceptual memory was biased toward the label. In addition, participants showed greater linguistic-bias when the target face was shown for a brief (rather than longer) period of time, making it more difficult to encode the physical structures of the faces (Fugate et al., 2018a, Experiment 3). In a similar study (Doyle and Lindquist, 2018), participants similarly showed greater perceptual memory biases toward learned (rather than target) affective faces when the faces were encoded with nonsense words. For example, even for familiar emotion categories (e.g., “anger”), participants who learned novel exemplars were more likely to indicate the learned face than an actual target face as long as that learned face was encoded with a word. These findings are consistent with early visual processing effects of words on perception, yet they do not test these effects directly. Indeed, some researchers have argued that perceptual memory tasks reflect reporting biases and have little to do with actual perception (see Firestone and Scholl, 2014).

According to the Theory of Constructed Emotion (Barrett, 2017b) (formally known as the Conceptual Act Theory, Barrett, 2006, 2011, 2012, 2013, 2014), perceivers integrate language (and other forms of top-down knowledge) to make sense of more generalized affective changes in a stimulus. That is, language (i.e., emotion words) helps to initiate predictive coding (Bayesian logic) which allows the person to make sense of a stimulus and to perceive it as belonging to a discrete category. This view is different from other theories of emotion, in which emotion perception is typically thought of as a bottom–up phenomenon (Tomkins, 1962, 1963; Izard, 1971, 1994; Ekman et al., 1972; Matsumoto et al., 2008; Brosch et al., 2010; Ekman and Cordaro, 2011). According to some of these views, language only plays a role in naming the emotional percept, but not in helping to create it in the first place.

The Theory of Constructed Emotion is consistent with the more general Label-Feedback Hypothesis (Lupyan, 2007, 2012; Lupyan et al., 2007; Lupyan and Clark, 2015). According to that theory, verbal labels play an active role in determining what an object is and how it should be categorized by augmenting top–down contextual knowledge (Lupyan and Ward, 2013; Lupyan and Clark, 2015). Consistent with this idea, Boutonnet and Lupyan (2015) found that participants who viewed label-cued pictures showed early and more positive P1 responses in the brain compared to participants cued with non-verbal sounds. Moreover, these neural responses predicted later behavioral responding (500 ms later) (Boutonnet and Lupyan, 2015). In a more recent study, verbal labels (compared to shapes) better constrained early visual processing of a shape of a stimulus (Noorman et al., 2018).

Both theoretical views are consistent with neural evidence showing that there are long-range connections in which information flows in both forward (feedforward) and backward (feedback) directions (see Lamme et al., 1998). In addition, top-down information can modulate reentrant, or feedback, pathways that convey higher order information to antecedent cortical areas. These connections contain a rich amount of information that contributes to a stimulus and can help build a stable representation of it (see a review by Gilbert and Li, 2013). These findings have led to a new understanding about the role and prevalence of top–down influences across the visual cortical hierarchy, including tasks of visual awareness and attention (Lamme, 2003; Gilbert and Li, 2013).

Despite the increasing neural and behavioral data supporting the role of language as a top–down source of knowledge important for directing visual processing, it remains unexplored whether emotion words can affect visual awareness and create attentional biases for emotional percepts. Therefore, the purpose of Experiments 1 and 2 was to test each, respectively.

## Experiment 1

Examining whether emotion words change which emotional face is available to visual awareness is key to understanding the role that language has on emotion perception in visual processing. Behaviorally, visual awareness is most commonly assessed with either binocular rivalry (BR) (Wheatstone, 1838) or continual flash suppression (CFS) techniques (Tsuchiya and Koch, 2005). In these paradigms, we use “visual awareness” to refer to the multitude of mechanisms that might determine what visual information is available to the visual system at a particular moment (e.g., Anderson et al., 2011a).

During BR, two different images are shown separately to each eye, such that they are unable to fuse in vision and undergo alternating periods of dominance and suppression (for a review of binocular rivalry, see Blake, 2001). Participants’ perception typically oscillates between one image and the other, as each stimulus competes for visual dominance. By measuring the amount of time that one of the two images is available to vision, it is possible to determine how the visual system selects the contents of visual awareness. Predominance is the total percentage of time that one of two images is available, and is calculated from the total percentage of time that one of two images is available divided by the total time either image is available. Predominance can come about in a number of ways, including: (1) an image remains dominant for longer periods of time, on average; (2) it remains suppressed for shorter periods of time, on average; or (c) it remains dominant for longer durations *and* suppressed for shorter durations. Sometimes blends of percepts do occur, and dominance can be calculated by dividing the total percentage of time that one of the two images is available by the total time either image and blends are available. Binocular rivalry likely involves multiple neural operations distributed over different stages of visual processing (e.g., Ooi and He, 1999; Blake, 2001; Nguyen et al., 2001), and involves both early and late processes (see also Sobel and Blake, 2002). It is also known to be modestly affected by selective attention, but that sustained training can alter predominance (Lack, 1978; Dieter et al., 2016). Theoretically, BR methodologies are often used to test a stimulus’s effect on visual awareness.

In one BR study assessing the role of previous written information on the visual awareness of emotion, structurally neutral faces previously associated with negative written information were available longer in visual awareness over structurally neutral faces previously associated with positive or neutral information (Anderson et al., 2011b). The results showed that negative written information can bias the visual awareness toward faces associated with a negative context.

Yet, there is no empirical evidence to assess whether an emotion word, on a trial-by-trial basis, can change the total time an emotional face is available to vision. In Experiment 1, we use BR to test whether emotion words affect which of two emotional faces is available for visual awareness on a trial-by-trial basis. To test the influence of emotion words on visual awareness, participants on each trial in Experiment 1 saw a control or an emotion word prior to two emotional faces presented under BR. Participants tracked the orientation of a gradient placed on each emotional face as an indirect judgment of which face they experienced during the ten second trial. We predicted that the emotional face congruent with the word prime would be available to visual awareness longer (compared to the time that face was available when a control word preceded the trial).

### Methods

#### Participants

Seventy (27 males, 43 females; ages: 18–22) undergraduates from Boston College participated for $10 cash or one research credit. We based our sample size on estimates for similar faces under similar conditions (e.g., Anderson et al., 2011a, 2012). All participants were native English speakers (or English birth-bilinguals) and had normal or corrected-normal vision. Participants either needed uncorrected vision or wore contact lens, since glasses interfered with the stereoscopic device. Participants signed a written and informed consent that was approved by the IRB from the affiliated University.

#### Stimuli

##### Emotional faces

**FIGURE 1:**
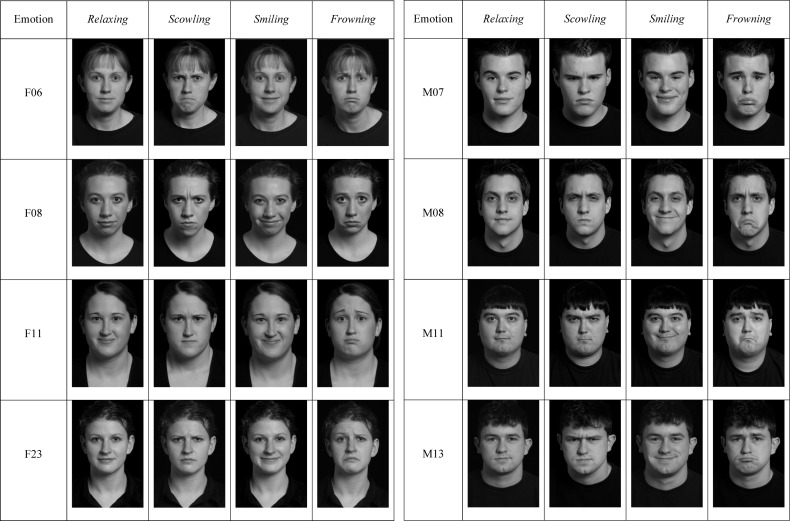
Stimuli used in Experiment 2 (includes stimuli for Experiment 1).

We selected *scowling, smiling, relaxing*, and *frowning* faces of the same identity from a standardized set of emotional faces created and maintained by the Interdisciplinary Affective Science Laboratory^1^. All faces were Caucasian, but both male and female identities were used. This face set has been externally validated and used in over 12 empirical, peer-reviewed and published studies. In addition, each face differed in arousal and valence and corresponded to one of the normative emotion categories of anger, calm, happy, or sad (*scowling*, anger: high arousal/negative; *smiling*, happy: high arousal/positive; *relaxing*, calm: low arousal/positive; *frowning*, sad: low arousal/negative). We used these four emotional faces from four individual identities (two male, two female identities) (see Figure 1). For each identity, we paired each emotional face with every other emotional face, across trials. Thus, there were six face pairs for each identity (*smiling-frowning, frowning-relaxing, frowning-scowling, scowling-smiling, scowling-relaxing, relaxing-smiling*). We used grayscale to present the faces. We presented each face pair with an individual face both on the right and on the left, across trials.

We added a small fixation circle (0.52 degrees) with black and white gratings over the nose of each face (see Bannerman et al., 2008). We created two copies of each face, one in which the gratings on the nose slanted to the left and one in which the gratings slanted to the right. Thus, in addition to counter-balancing the faces on the right and left in each face pair, we also counterbalanced the orientation of the gradient placed on each identity’s nose.

##### Word primes

We used four emotion words (“sad,” “angry,” “happy,” “calm”) as semantic primes, which corresponded to the normative emotion in the emotional faces (*frowning, scowling, smiling*, and *relaxing*), and two control words (“public,” “belief”). The control words represented non-emotional, abstract mental states and have been used in previous semantic priming studies (e.g., Fugate et al., 2018a, b).

#### Procedure

**FIGURE 2:**
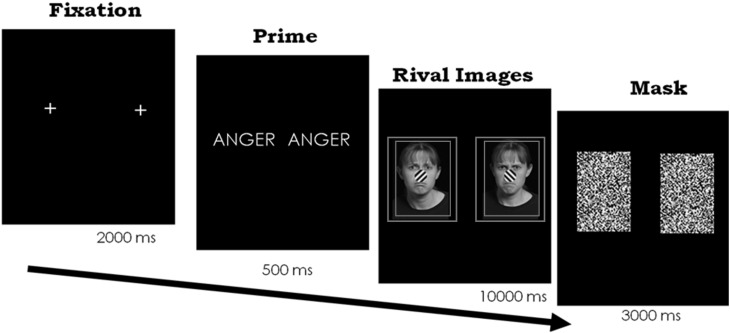
Paradigm and timing for Experiment 1. *Frowning* face on the left and *scowling face* on the right. In separate trials, the same two faces also followed the word “sad” and a control word. Each face appeared on the right and left, across trials. Stimuli images are from IAS Lab face set (http://www.affective-science.org) and depict adult participants from Boston College. Participants in this face set gave written and informed consent, including explicit consent to be photographed and their likeness to be reproduced.

We presented stimuli using E-prime (version 1, Psychology Software Tools Inc, 2002) on a Dell Optiplex 724 computer with a 17in Dell LCD flat-screen monitor (1280 × 1024). Participants sat with their heads in a chin rest and viewed the paired stimuli through a mirror stereoscope at approximately 55 cm. We presented the words and each face in the face pair so that they subtended approximately 1.8 × 1.4 degrees of the visual angle, which is large enough for a clear percept but also minimizes piecemeal rivalry^2^. On a given trial, participants first saw a fixation mark for 1000 ms, followed by either an emotion or control word for 500 ms to both eyes. Because we presented the fixation mark and the words to both eyes simultaneously, the image was fused in vision and the result was a normally looking word or fixation mark. Participants reported being able to read the word normally, as confirmed after the study’s completion (see below). Next, participants experienced the rivalrous emotional faces for a total of 10 s. Each face had a double-lined square placed around it to help with binocular fusion. Finally, participants saw a visual mask over the image in each eye for 3 s (see Figure 2).

Participants used their index finger on both hands to respond in rapid succession to the orientation of the line gradient they saw for the entirety of the trial. Participants used the “a” key to indicate the emotional face with the left-oriented gradient, and the “l” key to indicate the emotional face with the right-oriented gradient. We instructed participants to keep their index fingers on these two keys at all times and respond continually over the entirety of the trial. Participants pressed both keys if they could see both gratings at once, which sometimes occurs (see Meng and Tong, 2004; Alpers and Gerdes, 2007). We collected the time in which each key was pressed for each trial.

We presented each trial three times: twice with an emotion word (one matching the face on the right, and one matching the face on the left) and once with a control word. Therefore, the total number of trials was 288 [4 identities ^∗^ 6 face pairs ^∗^ 2 face locations (right vs. left) ^∗^ 2 gradient orientations (slanted right vs. left) ^∗^ 3 word primes].

#### Awareness Checks of Words

We asked participants to complete a questionnaire after the experiment to assess their awareness of the words used as primes (a retrospective measure of awareness). The questionnaire listed 28 words. Six of the words appeared and 22 did not appear. Participants circled the words they had seen and indicated their confidence in their answer. All participants had a *d*′ > 1.0, suggesting that they in fact saw (and remembered) which words were presented with high fidelity^3^.

#### Compliance to Direction

We asked participants after the experiment whether they were aware of seeing specific emotional faces and whether they felt they were able to track the orientation of the gradient on the faces continuously over each 10-s interval. No participant indicated that they saw faces which varied in emotion. One participant noted that they saw emotional faces but did not notice that the expression changed within a trial (only that there were different identities across trials). A large number of participants (*n* = 20) responded that they were not able (or did not feel confident that they were able) to track their vision continuously over the entire duration of each trial for the entirety of the experiment^4^. We removed these participants’ data. Therefore, the final number of participants in the data set was 50. Although a fair amount of participants’ data were removed, this is not entirely unexpected since participants were asked to report whether they felt they were able to continuously attend to rapidly changing stimuli over a 10 s interval for 288 trials. When we compared the removed participants’ data with those remaining participants’ data on the overall priming effect between emotion words and control words, there was no statistical difference in predominance (see below).

#### Data Coding

When the trial contained an emotion word, we added the total time a participant pressed the key associated with the right/left-oriented grating they experienced. When the trial contained a control word, we calculated the total time with respect to both emotional faces. We then calculated three ratios to provide predominance and dominance information. Predominance was defined as the percentage of time the word-consistent face was available divided by the total time that both faces were available. Dominance was defined as the percentage of time the word-consistent face was available divided by the total time both faces were available, including the time the participant saw both percepts together (e.g., blends). Whereas blends are often discarded and not considered in the analyses, analyzing both dominance and predominance ratios allowed us further insight into the mechanism by which emotion words affect binocular rivalry. That is, a high correlation between dominance and predominance ratios suggests that visual awareness under these conditions is affected more by dominance phases of rivalry than suppression phases. That is because when there are blends, nothing is suppressed. Therefore, if the pattern remains the same when blends are included or not included, the effect is likely due to dominance of one stimulus over the other. In addition, we calculated predominance scores by also including the “no response” key, which could be the result of a participant’s indecision, inability to comply with directions, or simple fatigue.

Although some researchers choose to report the first percept indicated in visual awareness, rather than predominance or dominance time, we followed Anderson et al. (2011a, 2012) methodology because top–down influences on perception are thought to affect the total time rather than the initial percept.

Next, we performed separate 2-factor repeated measures ANOVAs on each calculated ratio, using an alpha of 0.05. Factor one was the prime type (control word or emotion word), and factor two was the emotion word category (anger, calm, happy, sad). We followed-up any significant effects with dependent *t*-tests. We did not predict an overall effect of emotion word category, as there was no prior literature to suggest that there might be differences between the majority of pairs. The possible exception would be *scowling* faces which are often processed faster than other emotional faces, and therefore might be more likely to dominate over other emotional faces (Maratos, 2011). Therefore, we set our sample size to represent appropriate statistical power for the overall effect of prime type. Despite no overall prediction for an effect of emotion word category, we did find a significant or marginally significant effect for each ratio (see below). Therefore, we followed up each emotion word category separately for each ratio, but as the results show, individual follow-up tests for each emotion word are sometimes underpowered^5^. Finally, we performed a correlation on the three ratios to show their relationship.

### Results

We first assessed whether there was a difference among the individual identities and for when the faces were presented to the right or left eyes. No differences were found, *F*(3,51) = 0.48, *p* = 0.700, *t*(49) = −0.20, *p* = 0.842, respectively.

#### Predominance

Predominance ratios included the percentage of time the word-consistent face was seen exclusively divided by the total time both the faces were seen separately. No blends or periods of no responding were included. As predicted, we found a main effect of prime type, *F*(1,56) = 6.722, *p* = 0.006, one-tailed (Emotion word: *M* = 0.514, *SE* = 0.008; C.I. = 0.497–0.531) (Control word: *M* = 0.491, *SE* = 0.002; C.I. = 0.487–0.495), η_p_^2^ = 0.107, Power = 0.722. When participants saw an emotion word (compared to a control word), they experienced the congruent emotional face for approximately 2.3% longer. There was also a main effect of emotion category, *F*(3,168) = 4.220, *p* = *0.004*, one-tailed, η_p_^2^ = 0.070, Power = 0.852. The means and standard errors for each emotion category separately are shown in Table 1, along with the dependent *t*-tests between the emotion word and control word separately for each emotion category. The interaction between prime type and emotion category was not significant, *F*(3,168) = 0.889, *p* = 0.448.

**TABLE 1:** Predominance ratios for each of the four emotion categories and respective controls for Experiment 1.

**Emotion word**	**Mean (*SE*)**	**Control word**	**Mean (*SE*)**	**Dependent *t*-test**
Angry	0.503 (0.016)	Angry control	0.472 (0.014)	*t*(56) = 1.575, *p* = 0.061^¥^, *d* = 0.209
Calm	0.508 (0.012)	Calm control	0.503 (0.013)	*t*(56) = 0.581, *p* = 0.282, *d* = 0.077
Happy	0.545 (0.012)	Happy control	0.516 (0.008)	*t*(56) = 2.389, *p* = 0.010^∗^, *d* = 0.316
Sad	0.500 (0.011)	Sad control	0.474 (0.008)	*t*(56) = 2.007, *p* = 0.025^∗^, *d* = 0.266

*Paired *t*-tests are reported one-tailed, and were conducted on each of the four emotion word trials compared to the respective control word trials. ^∗^Significant at *p* < 0.05. *^¥^*Significant at *p* < 0.10. *d* is Cohen’s *d*.*

#### Dominance

Dominance ratios included the time participants reported seeing a blend (hitting both keys, simultaneously). On average, blends were seen roughly 17% of the time (1697 ms of 10,000). This number did not change whether the grating was to the left or the right for either key. Fourteen participants never reported blends; the remaining participants indicated average trial blend times ranging from 232 to 8495 ms (*M* = 1257 ms, *SE* = 246.99).

We found a marginally significant main effect of prime type, *F*(1,56) = 2.409, *p* = 0.063, one-tailed (Emotion word: *M* = 0.491, *SE* = 0.053; C.I. = 0.384–0.598) (Control word: *M* = 0.414, *SE* = 0.014; C.I. = 0.386–0.443), η_p_^2^ = 0.041, Power = 0.332. There was also a main effect of emotion category, *F*(3,168) = 2.128, *p* = 0.049, one-tailed, η_p_^2^ = 0.037, Power = 0.536. The means and standard errors for each emotion category separately are shown in Table 2, along with the dependent *t*-tests between the emotion word and control word for each emotion category separately. The interaction between these two factors was not significant, *F*(3,168) = 1.069, *p* = 0.364.

**TABLE 2:** Dominance ratios for each of the four emotion categories and respective controls for Experiment 1.

**Emotion Word**	**Mean (*SE*)**	**Control Word**	**Mean (*SE*)**	**Dependent *t*-test**
Angry	0.409 (0.016)	Angry control	0.369 (0.016)	*t*(56) = 2.130, *p* = −019^∗^, *d* = 0.282
Calm	0.420 (0.017)	Calm control	0.415 (0.017)	*t*(56) = 0.506, *p* = 0.308, *d* = 0.067
Happy	0.452 (0.017)	Happy control	0.409 (0.013)	*t*(56) = 3.242, *p* = 0.001^∗^, *d* = 0.429
Sad	0.684 (0.204)	Sad control	0.465 (0.038)	*t*(56) = 1.166, *p* = 0.124, *d* = 0.155

*Paired *t*-tests are reported one-tailed, and were conducted on each of the four emotion word trials compared to the respective control word trials. ^∗^Significant at *p* < 0.05. *d* is Cohen’s *d.**

#### Predominance Including No Key

Predominance ratios included the time participants reported not hitting a key, as well. Across trials types, every participant had some no key responses. Participants average trial “no response” times ranged from 539 to 9824 ms (*M* = 1582 ms, *SE* = 303.10).

We found a significant main effect of prime type, *F*(1,56) = 4.384, *p* = 0.021, one-tailed (Emotion word: *M* = 0.392, SE = 0.016; C.I. = 0.359–0.424) (Control word: *M* = 0.376, *SE* = 0.016; C.I. = 0.345–0.408), η_p_^2^ = 0.073, Power = 0.539. There was also a main effect of emotion category, *F*(3,168) = 5.060, *p* = 0.001, one-tailed, η_p_^2^ = 0.083, Power = 0.914. The means and standard errors for each emotion category separately are in Table 3, along with the dependent *t*-tests between the emotion word and control word for each emotion category separately. The interaction between these two factors was not significant, *F*(3,168) = 0.815, *p* = 0.487.

**TABLE 3:** Predominance ratios with “No Response” key for each of the four emotion categories and respective controls for Experiment 1.

**Emotion Word**	**Mean (*SE*)**	**Control Word**	**Mean (*SE*)**	**Dependent *t*-test**
ANGRY	0.377 (0.017)	ANGRY CONTROL	0.359 (0.018)	*t*(56) = 1.254, *p* = 0.108, *d* = 0.166
CALM	0.392 (0.018)	CALM CONTROL	0.388 (0.019)	*t*(56) = 0.421, *p* = 338, *d* = 0.056
HAPPY	0.413 (0.018)	HAPPY CONTROL	0.391 (0.017)	*t*(56) = 2.199, *p* = 0.016^∗^, *d* = 0.291
SAD	0.385 (0.017)	SAD CONTROL	0.367 (0.016)	*t*(56) = 1.797, *p* = 0.039^∗^, *d* = 0.238

*Paired *t*-tests are reported one-tailed, and were conducted on each of the four emotion word trials compared to the respective control word trials. ^∗^Significant at *p* < 0.05. *d* is Cohen’s *d*.*

#### Correlations Between Ratios

All three computed ratios were highly positively and significantly correlated, *r*^2^ (*n* = 51) = 0.539 between predominance and dominance scores, *r*^2^ (*n* = 51) = 0.477 between predominance and predominance with “no response” key included, and *r*^2^ (*n* = 51) = 0.780 between dominance and predominance with “no response” key included. Although the correlation is significant between dominance and predominance ratios, it is not perfectly so, suggesting that suppression might also play a substantial role in addition to dominance.

## Experiment 2

The results from Experiment 1 show emotion words play a role in the selection of material for visual awareness of emotion on a trial-by-trial basis. Although visual awareness is highly affected by attention, most researchers distinguish a difference between the two (see Lamme, 2003; Koch and Tsuchiya, 2007; Koivisto et al., 2009). In the case of Experiment 1, emotion words increased visual awareness of a congruent emotional face. Whether emotion words also affect selective visual attention is better addressed with divided visual field paradigms.

Divided visual field paradigms are common measures of attentional bias (MacLeod et al., 1986). Specifically, two stimuli are presented for a short time (∼200 ms), separated into two visual fields (Bourne, 2006). As such, information presented briefly to the right visual field (RVF) is initially processed by the left hemisphere, whereas information presented briefly to the left visual field (LVF) is initially processed by the right hemisphere. In Experiment 2, we examined the influence of emotion words on selective visual attention to emotional faces by using a DVF paradigm. Additionally, we used a divided visual field (DVF) paradigm to show that emotion words affect selective visual attention only for emotional faces presented in the RVF. This is because linguistic information presented to the RVF is processed primarily by the left hemisphere at the stage of processing (<200 ms) (Hellige, 1990).

Burt and Hausmann (2018) used a DVF paradigm to explore whether participants discriminated different emotional faces by referencing language. They showed that participants were better at discriminating between two emotional faces, but only when the target face was presented in the RVF compared to the LVF. Therefore, participants’ abilities to discriminate among category members were achieved by referencing linguistic knowledge.

In Experiment 2, to explicitly test the role of emotion words on the selective visual attention of emotional faces, we used a DVF paradigm in conjunction with the trial-by-trial semantic priming from Experiment 1. As a behavioral indicator of attention, we asked participants to make a simple non-linguistic determination about the location of a dot (e.g., dot-probe task). Latency to identify a dot is thought to be a sensitive measure of exogenous, bottom-up visual attention (e.g., Navon and Margalit, 1983; Lamme, 2003). We compared the time it took participants to indicate the dot when the trial was preceded with a congruent emotion word (to either the RVF face or the LVF face) versus a control word (see Methods for further detail).

We predicted that if emotion words (compared to control words) contributed to selective visual attention for emotional faces, then participants would be slower to detect a dot in the RVF that appeared in the same location as an emotional face congruent with the emotion word. We predicted that participants would be slower under these circumstances because the brain should integrate the emotion word with the congruent face, which evokes additional processing. As a result, participants would need to disengage from the additional linguistic processing as it contributed to the face in order to complete a non-linguistic task. As a consequence, participants should show a delayed response in these circumstances. For trials preceded by a control word, however, there should be no additional linguistic processing since the word meaning is not relevant to either face. In this case, participants’ reaction times should not be slowed (i.e., there is no need to disengage). Moreover, if the effects are truly linguistic in nature, they should be limited to the RVF. That is, there should be no difference in speed to detect the dot in the LVF because the right hemisphere does not engage in linguistic processing at this early stage (Wolford et al., 2000). Although we did not necessarily predict a difference in accuracy (since the task performance should be at or near ceiling), we also analyzed accuracy to make sure there was not a speed-accuracy tradeoff. For example, we wanted to ensure that the disengagement from the additional linguistic processing to the primed trials in the RVF was not accompanied by better accuracy, which could mean that participants were slower because they were more accurate. This would make the major finding difficult to interpret.

### Methods

#### Participants

Eighty-eight undergraduates (54 female, 34 male) from the University of Massachusetts - Dartmouth participated for one departmental research credit. One participant did not finish, one participant did not comply with directions, and another participant only completed one block of trials. In addition, five participants’ data were removed because their overall reaction times were statistical outliers. The total number of participants included in the final data analysis was 80.

We based our sample size on a combination of the effect size found for Experiment 1, as well as that for divided visual field studies of emotional faces (e.g., Burt and Hausmann, 2018).

All participants were native English speakers (or English birth-bilinguals) and had normal or corrected-normal vision. Participants signed a written and informed consent, which was approved by the IRB from the affiliated University. We restricted participants to those with normal or corrected-normal vision with contact lenses, as glasses interfered with the divided field paradigm. We also restricted participants to those who identified as right-hand dominant, as language is more likely to be left-lateralized in these individuals (Knecht et al., 2000).

#### Stimuli

##### Emotional faces

We used the faces from the same face set as in Experiment 1, but we included additional stimulus identities. We used four emotional faces (*scowling, smiling, relaxing*, and *frowning)* from each of four female and four male identities to create six emotional face pairs, as in Experiment 1 (see Figure 1).

##### Word primes

We used the same emotion words as in Experiment 1 (“angry,” “happy,” “calm,” and “sad”), but we added two additional control words so that each word appeared the same number of times across trials (“public,” “belief,” “honest,” and “space”).

#### Procedure

We presented stimuli using E-prime (version 2, Psychology Software Tools Inc, 2012) on a Dell Latitude E5570 laptop computer and a 15.6-inch LCD flat-screen monitor (1366 × 768).

Participants were required to use a central chinrest that ensured a consistent visual angle of 2.5° from the central fixation for the presentation of emotional faces. The chin rest also required that participants maintained a 45 cm distance from the computer screen. Both are necessary to ensure that participants only viewed the stimuli in a dichoptic manner. We placed a moist sponge at the base of the chinrest to help alleviate any eye dryness that might have occurred during prolonged gaze (see Bourne, 2006).

We first trained participants to keep their gaze focused on a central fixation cross without shifting their eyes throughout the experiment. To do that, the researcher sat next to the participant and watched his or her eyes for movement that were not blinks, but rather involved lateral movement. The experimenter watched the participant over a period of thirty practice trials, as has been done in other DVF studies (e.g., O’Hare et al., 2017).

Participants repeated the trials until they had fewer than two saccades overall and zero saccades in the last 30 s of the practice trials. The majority of participants (88%) required less than five practice sessions, with a median of two sessions. In the practice trials, participants were not asked to make responses and instead to try their best to focus their gaze on the fixation. The practice trials for saccades used different, non-emotional stimuli from the actual experiment. Once the participant passed the practice trials ensuring that saccades were no longer automatic, they responded to a short set of five practice trials with similar affective faces as those used in the actual experiment.

**FIGURE 3:**
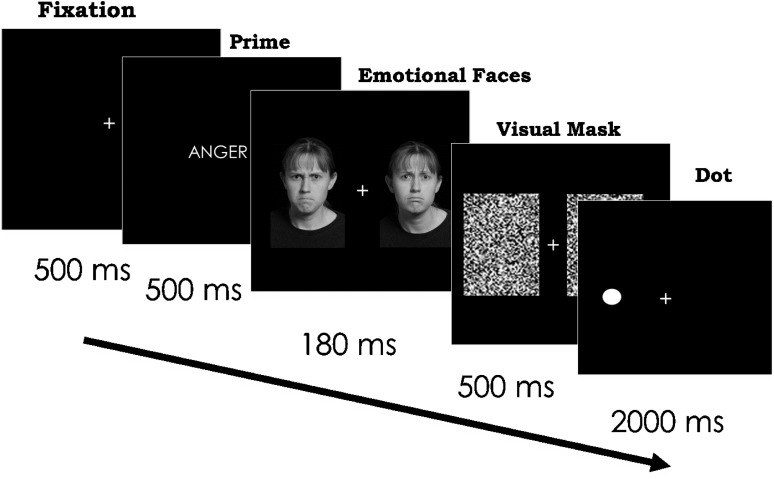
Paradigm and timing for Experiment 2. *Scowling face* on the left and *frowning* face on the right. In separate trials, the same two faces followed the word “sad” and a control word. Each face appeared on the right and left, across trials. In addition, the dot appeared on the right and left, across trials. Stimuli images are from IASLab face set (http://www.affective-science.org) and depict adult participants from Boston College. Participants in this face set gave written and informed consent, including explicit consent to be photographed and their likeness to be reproduced.

On each trial, we first presented a fixation cross for 500 ms. We next presented either an emotion word or a control word for 500 ms in the middle of the screen so that participants could fully read the word. Immediately after the presentation of the word, we presented two different emotional faces of the same identity simultaneously for 180 ms in each visual field (see Bourne, 2006). We presented the faces on the computer screen at a size of 4.33 × 6.47 visual degrees, 2.5 visual degrees from the center of the screen. Next, a visual mask appeared over the stimuli for 500 ms (see Figure 3). After the visual mask, participants saw a black dot in either the left or right visual space that was previously occupied by one of the emotional faces. The dot remained on the screen for 2000 ms. Participants were asked to identify the location of the dot in either the right or left visual field as quickly as they could without sacrificing accuracy. We collected accuracy and reaction time for each trial. Participants pressed the “d” key to indicate the presence of the dot in the LVF and the “k” key when the dot was in the RVF.

We presented each face in the face pair on the right and on the left, in separate trials.

Finally, we presented the dot for each face pair on the right and on the left, again across separate trials. We presented each trial combination three times: twice with an emotion word (one congruent with one face and one with the other face) and once with a control word. Therefore, the total number of trials was 576 (8 identities ^∗^ 6 face pairs ^∗^ 2 face locations (right vs. left visual field) ^∗^ 2 dot locations (right vs. left) ^∗^ 3 word primes).

#### Compliance to Direction

We did not assess word awareness in Experiment 2, since Experiment 1 suggested that participants were able to accurately detect and report on which emotion and control words were presented using similar semantic presentation times. We did not assess whether participants reported seeing any individual emotion face.

#### Data Coding

In order to assess the congruency between the emotion word and the face, we needed to first code the relationship between the two. From this, we could code whether the dot appeared on the same side as the emotional face congruent with the emotion word and whether it was in the RVF or the LVF. If the dot appeared in the same location as the emotion face that was congruent with the emotion word, it was considered the “primed” location. For example, imagine a trial in which the emotion word was “anger” and a *scowling* face appeared in the LVF and a *relaxing* face in the RVF. If the dot then appeared where the *scowling* face was previously located, we considered the dot to be on the “primed” side. If the dot appeared in the location of the *relaxing* face, however, we considered the dot to be on the “unprimed” side. Because each face pair was preceded by both emotion words (in different trials), the primed side and the unprimed side would switch when the emotion word was congruent with the *relaxing* face in the same pair. For example, given the same face pair as above but the emotion word was “calm,” the primed side would be where the *relaxing* face was previously located, and the unprimed side would be where the *scowling* face was previously located. Since control word trials had no congruency to either emotional face (e.g., “space” to a *scowling-relaxing* face pair), we had to code these trials with respect to both faces (as in Experiment 1). Then, for each emotion word, we could compare the time to locate the dot on either the primed or unprimed side compared to a trial which contained the same two faces but was preceded by a control word. Finally, we coded whether the primed location was in the RVF or the LVF.

### Results

To test our hypotheses, we performed a four-factor rmANOVA on reaction times to the dot location. Factor 1 was the prime type (emotion vs. control). Factor 2 was specific emotion word category (“happy,” “sad,” “calm,” and “angry”). Factor 3 was whether the dot was on the same side as the emotion-congruent face or not (primed vs. unprimed trials). Factor 4 was the visual field of the dot location (RVF vs. LVF).

**FIGURE 4:**
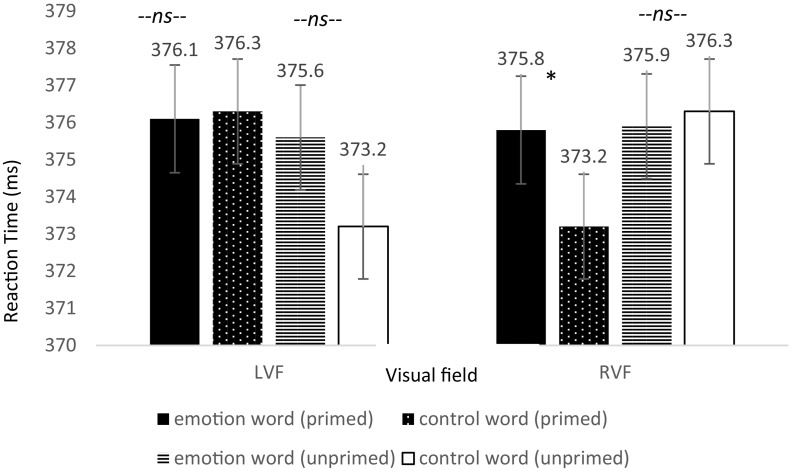
Reaction time differences between pair types by visual field for Experiment 2. There was a significant effect for the primed trials but only in the RVF (left-hemisphere) between emotion words and control words, in line with the hypothesis that participants would be slower to respond due to elaborative linguistic processing to trials preceded by an emotion word that is able to be integrated with the face.

Specifically, we predicted that if emotion words (compared to control words) contributed to the selective visual attention of emotional faces, then participants would be slower to detect the dot on “primed” trials when they occurred in the RVF. Thus, our hypothesis resided on a directional three-way interaction in which we expected a difference between emotion and control words for only the primed trials and only in the RVF. The omnibus three-way interaction between word type, primed trials, and visual field of the dot location was significant, *F*(3,237) = 2.83, *p* = 0.048, $\eta_{p}^{2}$ = 0.035, Power = 1.00^6^. Next we ran dependent t-tests between word type for primed trials appearing in the RVF and separately for those in the LVF. As predicted, only when the dot appeared in the RVF and the emotion face was primed did participants respond slower: *t*(79) = 1.883, *p* = 0.032, one-tailed, Cohen’s *d* = 0.210; Power = 0.525 (see Figure 4). Participants were 2.6 ms slower to respond to the dot in the RVF when it was primed with an emotion word compared to control word. Although these are small effects, their importance is contextualized when we consider the speed that the brain processes information. Importantly, there was no significant difference in the time for participants to locate the dot in the LVF under the same conditions, *t*(79) = −0.119, *p* = 0.453, one-tailed, Cohen’s *d* = 0.013. Furthermore, there was no significant difference in the time for participants to locate the dot in the RVF when the emotional face in that location was unprimed, *t*(79) = −0.223, *p* = 0.412, one-tailed, Cohen’s *d* = 0.02^7^.

**FIGURE 5:**
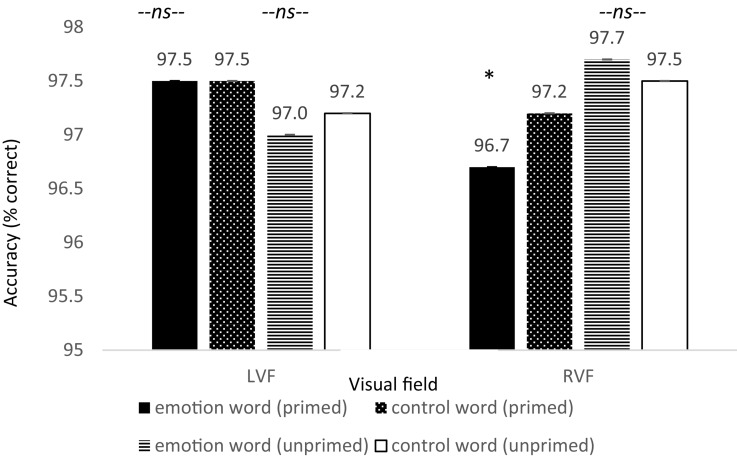
Reaction time differences between pair types by visual field for Experiment 2. There was a significant effect for the primed trials but only in the RVF (left-hemisphere) between emotion words and control words, in line with the hypothesis that participants would be slower to respond due to elaborative linguistic processing to trials preceded by an emotion word that is able to be integrated with the face.

In addition, there was a significant difference in accuracy between word types for primed trials appearing in the RVF, *t*(79) = −2.016, *p* = 0.024, Cohen’s *d* = 0.22, Power = 0.620, such that accuracy was lower on emotion word trials compared to control word trials. Moreover, there was no difference in accuracy for participants in the LVF under the same conditions, *t*(79) = −0.023, *p* = 0.981, Cohen’s *d* = 0.003, and no difference in accuracy for the unprimed trials in the RVF, *t*(79) = 0.414, *p* = 0.680, Cohen’s *d* = 0.092 (see Figure 5). Thus, the increase in reaction time for participants to locate the dot to primed trials in the RVF was not because participants were more accurate: rather their slower speeds were accompanied with worse accuracies^8^.

To specifically show that the effect was driven by a slowing down in processing and decrease in accuracy for emotion words (and not a change to the control words) under these conditions, we looked at the pattern between RT and accuracy for each condition between the emotion words and the control words (i.e., inverse efficiency score, Bruyer and Brysbaert, 2011). The RT-accuracy pattern was only significantly different between emotion and control word for the primed trials in the RVF, *p* < 0.005. There was no difference in the RT-accuracy pattern between the emotion and control words which were unprimed and in the RVF, primed and in the LVF, nor unprimed and in the LVF. This suggests that our significant three-way interaction was due to the emotion words slowing down processing and resulting in more errors, and not a speeding up and improvement of accuracy when there was a control word. Specifically, participants were slower and less accurate to detect the dot under these conditions because they were likely engaging in additional linguistic processing of the word-congruent face. Therefore, when visual attention was in the same location as the dot, the task was both more difficult and required more time to complete because participants needed to disengage from this additional linguistic processing. For trials preceded by a control word, there was no additional linguistic processing since the word meaning was not relevant, and therefore speed and accuracy were not affected. Likewise, neither was speed or accuracy affected in the LVF since the right-hemisphere does not engage in linguistic processing at this early stage (Wolford et al., 2000).

Moreover, the effect was limited to the RVF supporting our claim that the effects were linguistically modulated. Experiment 2 supports our hypothesis that emotion words affect selective visual attention.

## General Discussion

Our results are the first to show that emotion words serve as an important top–down influence on visual awareness (Experiment 1) and selective visual attention (Experiment 2) of emotional faces. Experiment 1 is among the first to use binocular rivalry to show that the meaning of a word can affect which of two highly controlled faces is available to the visual system. Whether this activation is by priming the emotion concept or affecting the emotional face directly, is yet to be explored. Other studies have shown that some stimuli dominate in visual awareness over other stimuli, but these effects can be attributed to gross structural differences or differences in low-level features among the stimuli rather than perceiver-based effects. Here we used tightly controlled face pairs (albeit varied face pairs using different identities depicting a variety of emotions) to assess the amount of time one face in a pair was available to awareness when primed with a congruent emotion or a control word. Specifically, in Experiment 1, when participants viewed an emotion word (vs. a control word) they perceived the word-congruent emotional face for longer. Emotion words (compared to control words) changed the duration that emotional faces were maintained in visual awareness, supporting our hypothesis.

In Experiment 2, we further explored the effect of emotion words on the processing of emotional faces by manipulating selective visual attention. Further, we examined whether the effects were limited to the RVF. Our effect was indeed limited to the RVF, consistent with the linguistic- processing advantage in the brain’s left hemisphere. Participants were slower to respond to the dot in the RVF when preceded by an emotion word congruent with the face in the same location compared to when primed with a control word. In addition, emotion words and control words presented to the LVF did not differentially affect participants’ reaction time to identify the location of a dot in the “primed” or “unprimed’ location. The slower reaction times were also accompanied by lowered accuracies, ruling out the possibility that participants might otherwise be sacrificing speed for accuracy. These findings suggest that when participants are presented with an emotion word followed by a congruent emotional face in the left hemisphere, endogenous selective attention is directed toward the continual processing of the emotion percept. Effectively, when the dot appears on the same side as an emotional face congruent with the emotion word, participants’ endogenous attention might create a type of “attentional blink” for the dot since they are engaged in processing of the word-face concept (Raymond et al., 1992). This is supported by the fact that the accuracies for the primed emotion word trials statistically differ between the RVF and the LVF. That is, participants are more accurate to the emotion word primed trials in the LVF than in the RVF.

The presence of an effect in the RVF (left hemisphere) supports the findings of Burt and Hausmann (2018). Their results showed that under normal conditions, participants integrate language in order to make categorical perceptions of emotional faces. Our results support the theory of cognitive penetration of perception and are consistent with both the Theory of Constructed Emotion (Barrett, 2017a), and the Label-Feedback Hypothesis (Lupyan, 2015). According to both theories, the brain is viewed as a predictive system that can incorporate top–down information (including familiarity with a word) at all perceptual stages to resolve prediction error to arrive at an optimal solution in the moment (see also Clark, 2013; Hutchinson and Barrett, 2019). As a result, predictions based on past experience influence relatively low-level perceptual processes and activity, including that within the primary visual cortex (see also den Ouden et al., 2012). In fact, the majority of synapses within primary visual cortex originate from top-down sources (see Sillito and Jones, 2002). Consistent with this idea, perception is facilitated when features of the internal model accurately anticipate the incoming stimulus, which creates perceptual fluency (Chanes et al., 2018).

Feedback projections from higher cortical areas directly and indirectly modulate neural activity in early visual areas (Webster et al., 1993, 1995). According to Bar and colleagues, the orbitofrontal cortex (OFC), a multi-modal brain region in the prefrontal cortex, is a prime candidate for such modulation (Bar, 2003; Kveraga et al., 2007). Specifically, they suggest that the OFC uses early incoming visual information from the dorsal stream to make a prediction about what the object might be. The OFC then sends this information to areas like the inferior temporal cortex (a region whose activation is associated with visual awareness), which updates the prediction with incoming bottom-up information until eventually the object is recognized and its affective significance registered (Kveraga et al., 2007; Barrett and Bar, 2009). The OFC is also connected with the amygdala as well as parts of the ventrolateral prefrontal cortex, including the inferior frontal gyrus that is important for semantic processing (for a review, see Binder et al., 2009). Through such connections, word meaning might influence what information is available to the visual system and/or is brought into awareness. Future studies should investigate whether this proposed mechanism is indeed the route by which words affect visual processing for emotional faces.

Our results are in line with an increasing amount of behavioral studies showing that language affects emotion perception at various stages of processing (Lindquist et al., 2006; Gendron et al., 2012; Doyle and Lindquist, 2018; Fugate et al., 2018a, b). Our findings are also consistent with a growing number of studies that show the role of language in the categorical perception of non-emotional (e.g., Gilbert et al., 2006; Drivonikou et al., 2007; Maier and Abdel Rahman, 2018), as well as emotional objects (for a review, see Fugate, 2013).

One limitation in Experiment 1 was that we did not include whether the last percept “overwent” the 10 s. That is, we recorded the total time up to the 10 s mark. It is possible that additional flipping might have occurred, affecting the total time, or that the last percept’s time was truncated. We do not expect that this would be systematic, however, and that any truncation would be randomly distributed across conditions. Although some have suggested that 10 s is a short trial time, we based this time off of previous similar research (Anderson et al., 2011a). Future studies might also look at what happens to visual awareness and selective attention when a fully or partially incongruent prime is used with respect to the emotion face. In a previous perceptual memory study using emotion word primes, some of the emotion words were either fully or partially incongruent with the face (Fugate et al., 2018a). These results showed that people’s memory was affected more by the label in these conditions.

### Implications

Our studies are the first to show that emotion words affect early visual processing, including visual awareness and selective visual attention. On a larger level, our results are consistent with a strong version of the Linguistic Relativity Hypothesis (Whorf, 1956): Words are integral to how people see and attend to information in the world in the first place (see also Gumperz and Levinson, 1996; Lucy, 1997; Wolff and Holmes, 2011).

## Author’s Note

All measures, manipulations, and exclusions in the studies are disclosed, as well as the method of determining the final sample size (with particular reference to whether collection was continued after data analysis). Portions of the data described in Experiment 1 were presented in poster format and symposium presentation at American Psychological Society conference in 2010 and later (2010) at a symposium presentation at the conference of the Society for Experimental and Social Psychology. Experiment 2 data was accepted as a poster presentation at the Society for Personality and Social Psychology in 2019. The authors adhered to APA ethical standards.

## Data Availability Statement

The datasets generated for this study are available on request to the corresponding author.

## Ethics Statement

Experiment was approved under IRB #10.051 from Boston College (2011) by Carolyn O’Connor, and Experiment 2 was approved under IRB #17.083 from the University of Massachusetts Dartmouth (2017) by A. Karburg.

## Author Contributions

JF conceived of the idea, with assistance from CM and AO’. JF collected and analyzed all data for Experiment 1. CM collected and analyzed all data for Experiment 2, with the assistance of JF. AO’ provided consultation for procedural demands in Experiment 2. CM and AO’ provided feedback to the manuscript. All authors approved the final manuscripts.

## Conflict of Interest

The authors declare that the research was conducted in the absence of any commercial or financial relationships that could be construed as a potential conflict of interest.

## Footnotes

1 Development of the Interdisciplinary Affective Science Laboratory (IASLab) Face Set was supported by the National Institutes of Health Director’s Pioneer Award (DP1OD003312) to Lisa Feldman Barrett. More information is available on-line at www.affective-science.org.

2 Piecemeal rivalry can occur when one experiences fluctuating patchworks of dominance consisting of jumbled portions from each eye’s view. Although Blake (2001) states that piecemeal rivalry can only be avoided with a much smaller visual angle, our dimensions were drawn from previous BR work of emotional stimuli (e.g., Anderson et al., 2011a).

3 A *d*’ of 1.0 is a somewhat liberal cutoff for awareness but we wanted to ensure that participants read (and remembered, since this is a retrospective measure) the specific emotion words they viewed.

4 Although 20 participants felt they were unable to pay attention and continually track their vision, only roughly one percent of the total trials were not answered (from ten different participants).

5 All statistical power values were calculated *post hoc*, using G^∗^Power, 3.1.9.2 (Faul, 1992–2014). We do not provide power analyses for non-statistical results.

6 There was also a significant interaction between emotion category, primed trials, and visual field, *F*(3,237) = 2.299, *p* = *0.039*, ηp2 = 0.028, Power = 0.379. When followed-up, however, emotion category was not significant. All other M.E.s and interactions were not statistically significant, *p*s > 0.05.

7 The dependent *t*-test on reaction times for emotion word primed trials between the right and the left visual fields was not significant, nor was the comparison for the emotion word unprimed trials between the right and the left, *t*(79) = −0.112, *p* = 0.911, and *t*(79) = 0.109, *p* = 0.914, respectively.

8 The dependent *t*-test on accuracy for emotion word primed trials between the right and the left visual fields was significant, *t*(70) = −1.876, *p* = 0.032, whereas the comparison for emotion words unprimed trials was not significant, *t*(79) = 1.503, *p* = 0.137.
